# Genome Characterization of a Novel Wastewater *Bacteroides fragilis* Bacteriophage (vB_BfrS_23) and its Host GB124

**DOI:** 10.3389/fmicb.2020.583378

**Published:** 2020-10-23

**Authors:** Mohammad A. Tariq, Fiona Newberry, Rik Haagmans, Catherine Booth, Tom Wileman, Lesley Hoyles, Martha R. J. Clokie, James Ebdon, Simon R. Carding

**Affiliations:** ^1^Gut Microbes and Health Research Programme, Quadram Institute Biosciences, Norwich Research Park, Norwich, United Kingdom; ^2^Norwich Medical School, University of East Anglia, Norwich, United Kingdom; ^3^Department of Biosciences, Nottingham Trent University, Nottingham, United Kingdom; ^4^Department of Genetics and Genome Biology, Leicester University, Leicester, United Kingdom; ^5^Environment and Public Health Research Group, School of Environment and Technology, University of Brighton, Brighton, United Kingdom

**Keywords:** bacteriophage, *Bacteroides fragilis*, genomes, waste water, human

## Abstract

*Bacteroides* spp. are part of the human intestinal microbiota but can under some circumstances become clinical pathogens. Phages are a potentially valuable therapeutic treatment option for many pathogens, but phage therapy for pathogenic *Bacteroides* spp. including *Bacteroides fragilis* is currently limited to three genome-sequenced phages. Here we describe the isolation from sewage wastewater and genome of a lytic phage, vB_BfrS_23, that infects and kills *B. fragilis* strain GB124. Transmission electron microscopy identified this phage as a member of the *Siphoviridae* family. The phage is stable when held at temperatures of 4 and 60°C for 1 h. It has a very narrow host range, only infecting one host from a panel of *B. fragilis* strains (*n* = 8). Whole-genome sequence analyses of vB_BfrS_23 determined it is double-stranded DNA phage and is circularly permuted, with a genome of 48,011 bp. The genome encodes 73 putative open reading frames. We also sequenced the host bacterium, *B. fragilis* GB124 (5.1 Mb), which has two plasmids of 43,923 and 4,138 bp. Although this phage is host specific, its isolation together with the detailed characterization of the host *B. fragilis* GB124 featured in this study represent a useful starting point from which to facilitate the future development of highly specific therapeutic agents. Furthermore, the phage could be a novel tool in determining water (and water reuse) treatment efficacy, and for identifying human fecal transmission pathways within contaminated environmental waters and foodstuffs.

## Introduction

Bacteriophages (phage) are viruses that infect bacterial cells and as a result can influence their growth, fitness and response to stress (Casjens and Hendrix, 2015; Davies et al., 2016). They are estimated to be numerically the most abundant biological entity on earth numbering at least 10^31^ (Hendrix et al., 1999; Bar-On et al., 2018). Phages are also a major constituent of the human microbiome and in particular, the intestinal microbiota where they can outnumber bacterial cells and human cells by up to 10:1 (Sender et al., 2016).

*Bacteroides* spp. are a dominant component of the intestinal bacteriome, accounting for between 5 and 40% of all anaerobes (Gorvitovskaia et al., 2016). In a recent study looking at 98 gut samples the relative abundance of *Bacteroides* spp. ranged from 0.37 to 98.82% (King et al., 2019). Although *Bacteroides fragilis* represents a smaller fraction of this group, it was present in all samples (King et al., 2019). *Bacteroides* spp. confer significant health benefits to their host including the digestion, processing and extraction of nutrients from complex plant-based polysaccharides, promoting colonic motility and angiogenesis, and the development of the gut-associated immune system (Hooper et al., 2003; Xu et al., 2003; Mazmanian et al., 2005).

*Bacteroides* spp. are also important clinical pathogens and can contribute to anaerobic infections (Perez-Brocal et al., 2015). *B. fragilis* is one of the most commonly isolated anaerobic pathogens from soft tissue infections and bacteremia (Shenoy et al., 2017). The capsular polysaccharide complex of *B. fragilis* consisting of two distinct polysaccharides is the primary mediator of intra-abdominal abscess formation (Tzianabos et al., 1993). Enterotoxigenic *B. fragilis* also produces metalloprotease toxins (fragilysin), which unlike pore-forming toxins, breakdown connective tissue through proinflammatory cytokine signaling leading to an increase in the permeability of the epithelial barrier, causing diarrheal diseases and acute inflammation (Wu et al., 2004; Kim et al., 2006).

Anaerobic bacteria and their phages have been proposed as candidates for indicators of fecal pollution as they do not replicate in estuarine waters and the phages are resistant to chlorine inactivation (Booth et al., 1979; Tartera and Jofre, 1987; McMinn et al., 2014, 2017; Dias et al., 2018). Despite the clinical importance of *B. fragilis*, and a study reporting up to 3 × 10^4^ PFU/100 mL of *B. fragilis* phages in sewage influent (Sun et al., 1997), only three complete genomes of *B. fragilis* phages have been described to date, two of which have been published with the third deposited under accession MN078104; all are virulent phages (Puig and Girones, 1999; Hawkins et al., 2008; Ogilvie et al., 2012). Here we describe the isolation of a new *B. fragilis* phage, vB_BfrS_23 from municipal wastewater and detail its genome characteristics. We also sequenced the host bacterium *B. fragilis* strain GB124 isolated from a United Kingdom municipal wastewater sample (Payan et al., 2005).

## Materials and Methods

### Bacterial Culture and Growth Conditions

*Bacteroides fragilis* GB124 was used as the host reference strain for phage isolation and has been used to detect human fecal contamination in water sources (Payan et al., 2005; Ebdon et al., 2007), and to test the treatment efficacy of water reuse technologies (Purnell et al., 2015, 2016; Dias et al., 2018). *Bacteroides* phage recovery medium (BPRM) was used to cultivate host GB124 and propagate the phage (Supplementary Table 1).

### Phage Isolation and Purification

Phage vB_BfrS_23 was isolated from 100 mL raw (untreated) municipal wastewater from a United Kingdom-based treatment plant. Wastewater was filtered with a 0.45 μm PES membrane syringe filter (Sartorius UK Ltd.) and concentrated by centrifugation for 15 min at 5,000 *g* using Amicon Ultra-15 10K centrifugal filter units. One milliliter of this concentrated sewage filtrate was mixed with 1 mL of mid-exponential growth phase (OD_620__nm_ 0.3–0.4) *B. fragilis* GB124 allowing 5 min for adsorption and was then added to semi-soft BPRM agar (0.35%) and poured on BPRM agar (1.5%) (Supplementary Table 1; Ebdon et al., 2007). After 18 h anaerobic incubation (5% CO_2_, 5% H_2_ and 90% N at 37°C and ∼25 psi pressure) the plates were screened for plaques. A single plaque was picked using a sterile pipette and resuspended in 10 mL BPRM medium containing sub-cultured host (OD_620__nm_ 0.3–0.4). The suspension was incubated for 18 h to allow further propagation of the phages. The sample was filtered through a 0.22 μM PES membrane filter (Sartorius UK Ltd.). The procedure was repeated a further three times to obtain a pure phage stock. This stock was used to further propagate and increase the phage titer. Fifty microliter was used to perform serial dilutions and was added to semi-soft BPRM agar (0.35%) with 200 μL of mid-log phase (OD_620__nm_ 0.3–0.4) bacterial host. The plates were incubated for 16 h in an anaerobic cabinet (5% CO_2_, 5% H_2_ and 90% N at 37°C and ∼25 psi pressure). Five milliliter SM buffer (100 mM NaCl, 8.1 mM MgSO_4_.7H_2_O and 50 mM Tris.HCl pH 7.4) was added to a plate of complete cell lysis and left on a mini gyratory shaker SSM3 (Stuart, United Kingdom) for 1 h. The top agar was harvested along with the buffer and transferred to a 50 mL tube (Corning, United Kingdom), after a brief vortex the tube was centrifuged at 3,000 × *g* for 10 min and the supernatant filtered through a PES membrane bottle top vacuum filter using ∼100 psi pressure (Millipore Millivac, Merck UK). The titer was evaluated using dilutions 10^–1^ to 10^–9^ and the titer adjusted to 1 × 10^8^ PFU/mL for temperature assays and was stored at 4°C for the duration of the experiments.

### Transmission Electron Microscopy

Briefly, a small drop of phage suspension containing ∼1 × 10^7^ PFU/mL was applied to a formvar/carbon coated copper transmission electron microscopy (TEM) grid (Agar Scientific, Stansted, United Kingdom) and left for 1 min. Excess liquid was removed with filter paper. A small drop of 2% uranyl acetate (BDH 10288) was applied to the grid surface and left for a further 1 min after which it was removed with filter paper. Grids were left to thoroughly dry before viewing and imaging using a Talos F200c TEM with Gatan Oneview digital camera.

### Host Range Assay

In total, eight *B. fragilis* strains (Supplementary Table 2) were used to determine the host range and specificity of vB_BfrS_23. Bacterial strains were cultured in BPRM broth to exponential phase (OD_620_ 0.3–0.33) prior to incorporation into double BPRM agar overlays (Ebdon et al., 2007). Dilutions of vB_BfrS_23 were then spotted onto the double agar overlay and observed for plaques following 16 h in an anaerobic cabinet (5% CO_2_, 5% H_2_ and 90% N at 37°C and ∼25 psi pressure).

### One-Step Growth Curve and Eclipse Period

To determine the burst size and latency period of vB_BfrS_23, a one-step growth curve was carried out (Kropinski, 2018). Initially, 9.9 mL of *B. fragilis* GB124 was grown anaerobically (5% CO_2_, 5% H_2_ and 90% N at 37°C and ∼25 psi pressure) to mid-exponential phase and 0.5 OD_620_. One-hundred μL of 1 × 10^7^ PFU/mL phage was then added for 5 min to allow phage adsorption. A 0.1 mL aliquot was then used to make ten-fold serial dilutions to a final dilution of 1 × 10^1^. As an adsorption control, a 1 mL aliquot from the 1 × 10^3^ dilution flask aliquot was added to 50 μL of CHCl_3_ and kept on ice for the duration of the experiment (less than 4 h). At various time points 0.1 mL was taken from each dilution and mixed with 200 μL of bacterial host suspension (in BPRM broth) and plated using 0.35% (w/v) BPRM agar. The data were normalized by multiplying the adsorption control and the value obtained from 1 × 10^3^ PFU/mL flask by x10, 1 × 10^2^ PFU/mL flask by x100 and 1 × 10^1^ PFU/mL flask by x1000. The burst size was determined as previously described (Kropinski, 2018).

At each sampling point 475 μL was taken to determine the eclipse period. The sample was added to 25 μL of chloroform (5%v/v), vortexed for 10 s and kept on ice until the end of the experiment to allow the chloroform to settle. One hundred microliter was taken from each timepoint sample and added to 200 μL of bacterial host and plated using 0.35% (w/v) BPRM agar. The plates were incubated for 16 h in an anaerobic cabinet (5% CO2, 5% H2 and 90% N at 37°C and ∼25 psi pressure). Each one-step growth and eclipse experiment were repeated to give three biological replicates.

### Thermal Assay

The viability of vB_BfrS_23 at different temperatures was assessed by incubating phage preparations of known titers at 4, 24, 30, 37, 40, 45, 60, 70, or 80°C for 15, 30, or 60 min out of direct sunlight. Serial dilutions of the phage stocks were incubated with 200 μL of bacterial host culture in BPRM broth for 15 min at 37°C prior to mixing with 5 mL BPRM semi-soft agar (0.35%, w/v) and pouring onto BPRM agar plates and incubated for 18 h at 37°C in anaerobic cabinet (5% CO_2_, 5% H_2_ and 90% N at 37°C and ∼25 psi pressure). For accuracy, plaques were counted on plates containing between 30 and 300 plaques.

### DNA Extraction

For Illumina sequencing, phage preparations (∼10^9^ PFU/mL) were incubated with RNase A (100 U Ambion) and Turbo DNase (2U Invitrogen) at 37°C for 30 min to remove bacterial chromosomal DNA. Nucleases were heat-inactivated at 65°C in the presence of 15 mM EDTA for 10min. The Norgen Phage DNA isolation kit (Geneflow Limited, Lichfield, United Kingdom) was used to extract the phage DNA. For Nanopore sequencing, phage was PEG-precipitated (10% (w/v) PEG 8000 and 6% (w/v) NaCl), treated with DNase (4 U Turbo DNase; Invitrogen) and RNase A (100 U; Ambion) followed by treatment with SDS (0.5%, w/v) and 4 μL (80 μg of proteinase K 20 mg/mL, Ambion) treatment at 55°C for 1 h and heat inactivation at 75°C for 15 min. Lipids and proteins were removed by mixing the sample 1:1 with chloroform and vigorous shaking for a few seconds followed by centrifugation at 15,000 *g* at 20°C for 5 min. The upper aqueous phase was treated with NaCl (0.2 M final concentration) prior to mixing 1:1 with isopropanol and left in -20°C for 16 h. The sample was centrifuged at 13,000 *g* at 20°C for 1 h followed by two washes with 70% ethanol prior to resuspending the pellet in nuclease-free water (Invitrogen). Bacterial DNA was extracted from an overnight culture of GB124 grown in BPRM broth, the sample was centrifuged at 3,000 *g* for 20 min, the pellet was resuspended using 300 μL of TE buffer in accordance to the Promega Maxwell^®^ RSC Cultured Cell DNA Kit (AS1620) protocol (FB211) and run on the Promega Maxwell^®^ RSC Instrument (AS4500).

### DNA Sequencing

Phage and bacterial genomic DNA were sequenced using Illumina and MinION ONT sequencing platforms. For MinION sequencing, the standard ONT protocol and native barcoding kit EXP-NBD104 with the ligation sequencing kit SQK-LSK109 were used. In brief, 1 μg of high quality vB_BfrS_23 and *B. fragilis* GB124 DNA was end-repaired and dA-tailed using the NEBNext FFPE Repair Mix (M6630) and NEBNext End Repair/dA-tailing (E7546). The native barcode (EXP-NBD104 kit) was used to barcode the samples and they were ligated using NEB Blunt/TA Ligase Master Mix (M0367). The sequence adapters were ligated with NEBNext Quick Ligation Module (E6056) and the samples were primed and loaded using the Flow Cell Priming Kit (EXP-FLP001) on the MinION R9 4.1 FLO-MIN106. Samples were run for 72 h, and the raw reads were base-called using Guppy v3.5.1.^1^ Adapters were removed using Porechop v0.2.3 (rrwick/Porechop, 2020).^2^ Genomic bacterial DNA was also sequenced using the Illumina MiSeq system. Briefly, the Illumina Nextera XT (Illumina, Saffron Walden, United Kingdom) library preparation kit was used to prepare sequencing libraries prior to running on Illumina MiSeq 2 × 150-cycle v2 chemistry. Paired-end sequencing reads were provided as FASTQ files with the raw reads having their adapters removed using Trimmomatic (Bolger et al., 2014) prior to quality trimming using Sickle at –q 30 and –l 15 (Joshi and Fass, 2011).

### Phage Genome Assembly and Annotation

Illumina MiSeq and MinION generated sequences were assembled using Unicycler v0.4.8 (Wick et al., 2017) resulting in a single contiguous circular sequence of 48,011 bp. The genome was annotated using RAST (Aziz et al., 2008; Overbeek et al., 2014; Brettin et al., 2015). The putative functions of the coding regions (CDS) were predicted using NCBI-nr (June 15, 2020) and Conserved Domain Database (CDD) (June 15, 2020) searches using BlastP and tBlastn. For Blastp and tBlastn, hits were considered significant if the e-values were lower than 1 e^–5^ at ≥60% protein identity (Altschul et al., 1990). For CDD searches, only hits with an e-value of 0.01 or lower were considered significant (Marchler-Bauer et al., 2015).

### GB124 Genome Assembly, Quality Checks and Annotation

The genome was assembled using Illumina MiSeq and ONT MinION reads, using Unicycler (Wick et al., 2017). Following host assembly, the contig was annotated using Prokka v.1.14.6 (Seemann, 2014). Antimicrobial resistance genes were investigated with ABRicate v.0.9.8 (tseemann/abricate, 2020)^3^ using Resfinder v3.2 (database version September 10, 2019) (Zankari et al., 2012), NCBI and AMRFinderPlus v3.8 (database version 2020-05-04.1) (Feldgarden et al., 2020). Insertion elements were predicted using ISfinder (Siguier et al., 2006).^4^ Significant hits (Score > 100 and e value <4e-11) in ISfinder were examined in the Prokka GenBank file and protein sequence submitted to BlastP. Suspected insertion sequence (IS) elements were visualized in Artemis 18.1.0 (Carver et al., 2012) and investigated for downstream Anti-Microbial Resistance (antimicrobial resistance) genes. ABRicate hits were considered significant if the coverage and identity were >90%.

Plasmids were identified using the PLSDB web server^5^ (data v2020_03_04) and coding regions found using Prokka v1.14.6 (Seemann, 2014; Galata et al., 2019). The putative functions assigned by Prokka were checked using BlastP according to NCBI-nr (July 1, 2020) and Conserved Domain Database (July 1, 2020) (Altschul et al., 1990; Marchler-Bauer et al., 2015). Hits were considered significant if the e-values were lower than 1e^–^5 at ≥ 80% protein identity. Plasmids were visualized using Brig v0.95 (Alikhan et al., 2011). The plasmids were screened for antimicrobial resistance and virulence genes using ABRicate v.0.9.8 and Resfinder, NCBI AMRFinderPlus and VFDB (Zankari et al., 2012; Chen et al., 2016; Feldgarden et al., 2020). The completeness and contamination of the seven-contig assembly were assessed using CheckM v1.0.18 (Parks et al., 2015). Average nucleotide identity with the type strain of *B. fragilis* was assessed using fastANI v1.2 (Jain et al., 2018) and *B. fragilis* CCUG 4856^T^ (RefSeq assembly accession GCF_005706655).

### vB_BfrS_23 Phage Genome Comparison of Large Terminase Subunit and Tail Fiber

The coding region for the genes were tblastx searched using default parameter and amino acid sequences sharing identity to the large terminase subunit and the tail fiber sequences were aligned using MAFFT v7 (Katoh and Standley, 2013). The L-INS-i algorithm with default parameters was used to improve accuracy. The alignment file was used to create a p-distance analysis in MEGA7 (Kumar et al., 2016) following construction of a neighbor-joining tree on p-distance using 1,000 bootstrap analyses using default parameters (Saitou and Nei, 1987).

### vB_BfrS_23 and ϕB124-14 Linear Genome Comparison Alignment

A detailed comparison of the vB_BfrS_23 with ϕ124-14 (Ogilvie et al., 2012), B40-8 and *Bacteroides* phage Barc2635 was performed using tBLASTx in Easyfig (Sullivan et al., 2011). The annotated GenBank file of vB_BfrS_23 was compared with the GenBank file for ϕB124-14, B40-8 and *Bacteroides* phage Barc2635.

## Results

### Phage Isolation and Phenotypic Characterization

**FIGURE 1:**
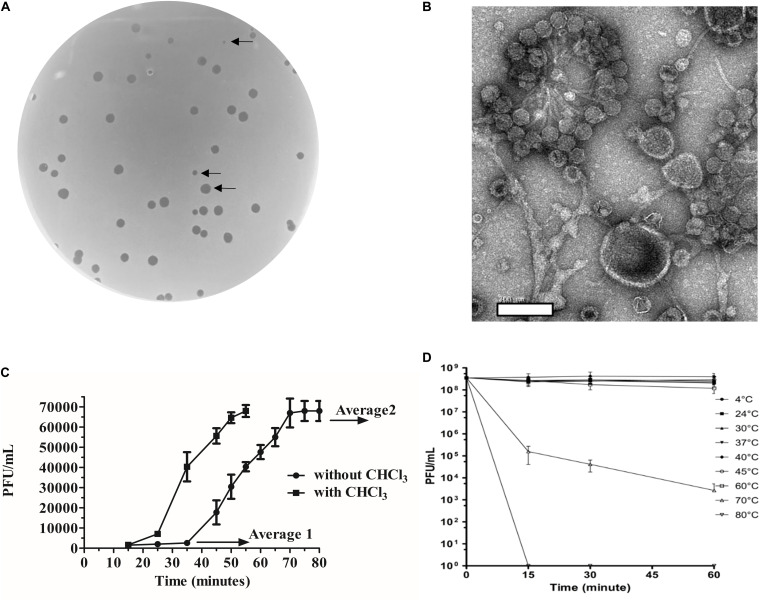
Morphological and biological characteristics of vB_BfrS_23. **(A)** Plaques of vB_BfrS_23D seen on a lawn of *B. fragilis* GB124. Arrows identify plaques of different sizes (2mm to 0.5mm). **(B)** Negatively stained TEM images of vB_Bfr_23D. Scale bar = 200nm. **(C)** One-step growth curve: The latent and rise periods were determined to be approximately 37 and 30 min, respectively, with a rise period and a burst size per cell of approximately 44 phage/cell (mean of 3 replicates). The Average 1 value is the mean PFU/mL of three time points before the rise period with the Average 2 value being the mean PFU/mL of three time point after the one step burst. The error bars depict SEM values (n = 3 biological replicates). The eclipse period was determined to be about 23 min. **(D)** Temperature assay: The vB_BfrS_23 phage was stable up to 60°C, with declining viability at higher temperatures after 1 h incubation. The error bars depict SEM values (*n* = 3 biological replicates).

*Bacteroides fragilis* GB124 was used as a host for phage discovery and the starting point for the screening of a filtered raw wastewater sample. We identified and isolated a virulent phage capable of infecting GB124, that generated plaques that ranged in size between 0.5 and 2 mm (Figure 1A). TEM images revealed the presence of a non-contractile long tail ∼150 nm and an icosahedral head ∼50 nm in size consistent with vB_BfrS_23 belonging to the *Siphoviridae* family of the order *Caudovirales* (Figure 1B).

### vB_BfrS_23 Phage Characteristics

Phage vB_BfrS_23 was seen to infect and lyse only one of the eight *B. fragilis* strains tested, GB124, which was the host strain used for isolating vB_BfrS_23 (Supplementary Table 2).

The one-step growth curve experiment (Figure 1C) showed that the phage had a burst size of ∼44 phage/cell (mean of three independent experiments) and latency period of ∼37 min. The eclipse period was determined to be ∼23 min (*n* = 3). It was also stable at temperatures between 4°C and 60°C (Figure 1D) with viability decreasing at 60°C with a more rapid inactivation seen at 70°C. No plaques were seen at 80°C. At 37°C plaques were of sizes up to 2 mm (Supplementary Figure 7). Interestingly, a slight increase in PFU/mL was seen between 40°C and 45°C, with the plaques being more uniform and smaller (0.5 mm) at 45°C (Supplementary Figure 7).

### Phage Genome and Phylogeny

**FIGURE 2:**
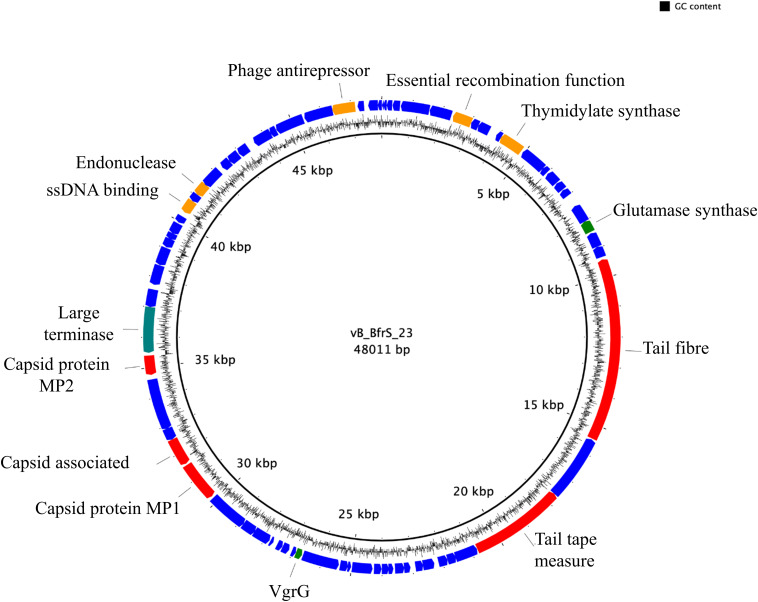
Genome map of vB_BfrS_23. The prediction and direction of the coding regions are indicated by arrow heads. The blue coding regions are hypothetical proteins with no known function. The 13 putatively known functions of the phage are annotated. Also shown are genes with predicted roles in replication and regulation (orange), DNA packaging (green), and structure (red).

**FIGURE 3:**
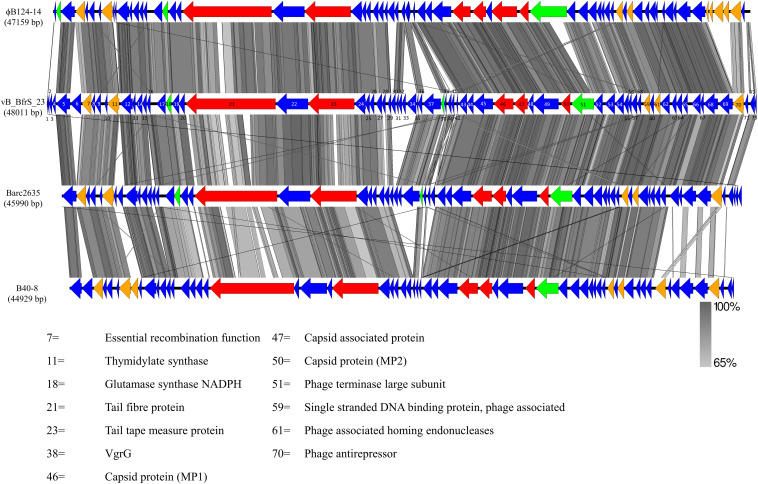
Genome comparison of vB_BfrS_23, Barc2635, ϕB124-14, and B40-8. Sequence similarity is represented by the gray scale bar. Blue colored regions are hypothetical proteins with no known function. Genes with predicted role in replication and regulation (orange), DNA packaging terminase (green) and structural (red) are also shown. The coding sequences (CDS) for vB_BfrS_23 are numbered and represented by directional arrows. CDS with putative function are listed and the numbers are linked to the CDS of vB_BfrS_23.

vB_BfrS_23 is a double-stranded DNA phage of 48,011 bp with a GC content of 38.6%, containing 73 putative CDS (Figure 2 and Supplementary Table 3). It was most similar to the virulent phage ϕB124-14 (86% query coverage) followed by *Bacteroides* phage Barc2635 (85% query coverage) and then ϕB40-8 (73% query coverage). The linear genome comparison of the vB_BfrS_23, Barc2635, ϕB124-14 and ϕB40-8 phages is illustrated in Figure 3.

**FIGURE 4:**
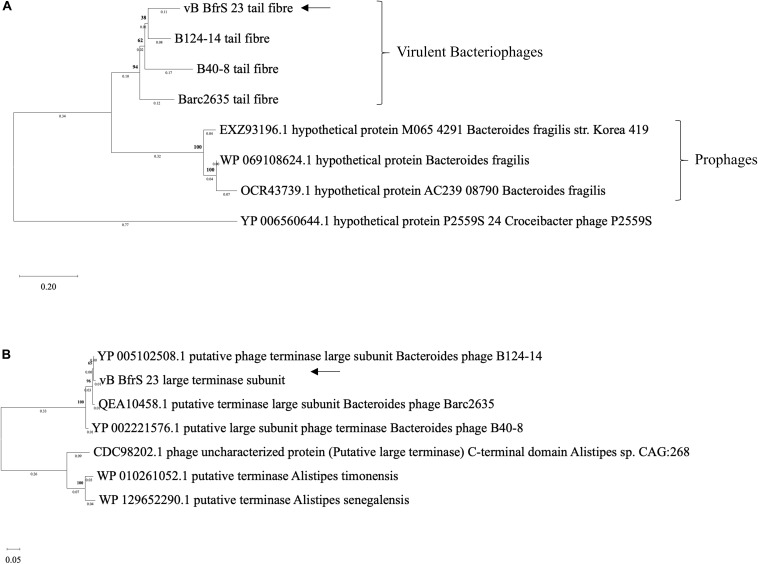
Phylogenetic comparison of vB_BfrS_23. Amino acid sequences of tail fiber proteins **(A)** and large terminase subunit proteins **(B)** were aligned using MAFFT v7 the L-INS-i algorithm with default parameters used to improve accuracy with the p-distance used to generate a neighbor joining tree with 1000 bootstrap replicate. The arrows depict vB_BfrS_23 location in the tree. The bold number above the node show the percentage bootstrap. The sum of the tree length in panel **(A)** is 2.17 and 0.87 in panel **(B)**.

The terminase large subunit and the tail fiber proteins were used to generate a phylogenetic tree (Figure 4). Both the tail fiber (Figure 4A) and terminase large subunit (Figure 4B) were shown to be most similar to ϕB124-14. BlastP^6^ revealed 13 of the CDS had a putative function and 8 CDS contained conserved domain signatures. Most of the CDS were assignable to genome structure and replication/regulation, with the remainder associated with lysis and DNA structure. Putative CDS of similar function clustered together to form modules. However, 6 putative proteins identified in phage ϕB124-14 were not found within the vB_BfrS_23 genome (Ogilvie et al., 2012). Ten putative CDS showed no homology to any protein within the database, with 27 sharing the highest sequence similarity to genes in ϕB124-14, 27 to Barc2635 and 8 to ϕB40-8 (the following to prophage regions) and 1 to a *Bacteroides ovatus* phage (Supplementary Table 3).

Like ϕB124-14, Barc2635 and ϕB40-8, vB_BfrS_23 lacked an obvious virulent genome module and only contained 1 putative protein that alluded to a strictly lytic life cycle (CD 18). CD18 exhibited closest homology to a putative peptidase protein identified within ϕB124-14 and contained a peptidase superfamily domain. The peptidase protein appeared to reside within a cluster of unassigned proteins, suggesting it may be a putative virulent module.

Five CDs were assigned a predicted function relating to virus replication and regulation. CD11 encoded a putative thymidylate synthase, which is a key enzyme in the synthesis of 2′-deoxythymidine-5′-monophosphate, an essential precursor for DNA replication. A conserved domain region identified within the protein suggested it encodes a ThyA-like enzyme as reported in the ϕB124-14 genome (Ogilvie et al., 2012). CD7 (recombination protein) and CD70 (anti-repressor) were also encoded within the replication and regulation genome module, promoting transcription of phage genes (Lemire et al., 2011).

### *B. fragilis* GB124 Genome Assembly and Annotation

**FIGURE 5:**
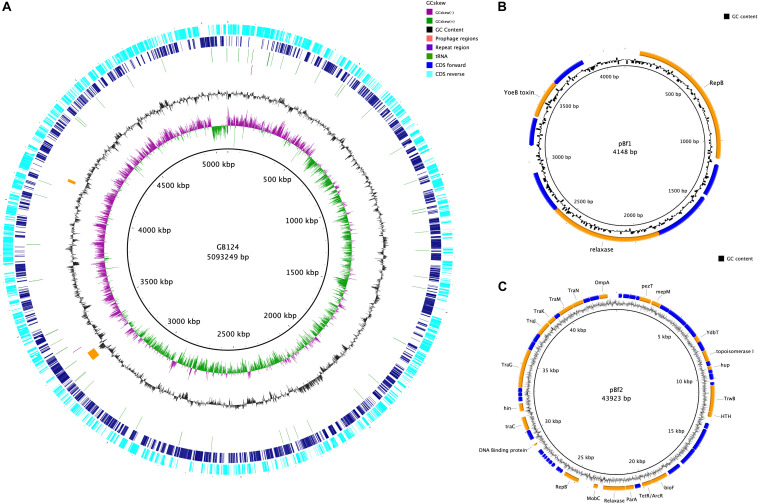
Genome map of *B. fragilis* GB124 and its plasmids. **(A)** The outer most ring depicts the coding sequences (CDS) in both anti-clockwise (aqua) and clockwise (blue) orientation. tRNAs are depicted as green arcs with the purple arcs depicting 2 CRISPR repeat regions. The orange arcs depict two prophage regions with by GC content shown as black and GC skewing by green and purple. **(B)** Circular map of plasmid 1 (S4). The blue coding regions are hypothetical proteins with no known function. The orange coding regions highlights a putative function. **(C)** Circular map of plasmid 2 (S5) using the same color coding as in panel **(B)**.

The genome was assembled into 7 contigs of >100 bp (N50 4,986,460 bp). CheckM analysis showed the genome to be 99.26% complete with no contamination. It shared 99.03% average nucleotide identity with *B. fragilis* CCUG 4856^T^, confirming GB124 as an authentic strain of *B. fragilis* (Chun et al., 2018). Two contigs were complete assemblies for plasmids which were identified using PLSDB and were named PBf1 and PBf2. PBf1 consisted of 4148 bp, was circular and contained eight predicted open reading frames (ORFs; Figures 5A–C and Supplementary Figure 4). PLSDB revealed an exact match to *B. xylanisolvens* strain H207 plasmid unnamed2 (NZ_CP041232.1). A further Blastn search showed a 100% identity and query cover match to one other plasmid, *B. ovatus* strain 3725 D1 iv plasmid unnamed3. PBf1 contained a YoeB toxin, a toxic component of a type II toxin-antitoxin system that helps to maintain plasmid stability by post-segregation killing or genetic addition (Figure 5B and Supplementary Figure 5) (Gerdes et al., 1986; Yarmolinsky, 1995). PBf2 consisted of 43,923 bp, was circular and contained 57 coding regions (5 domains of unknown function, 30 hypothetical proteins and 22 of putative function) (Figure 5C). A PLSDB search revealed 2 hits: *B. ovatus* strain 3725 D1 iv plasmid unnamed2 (NZ_CP041397.1) and *B. thetaiotaomicron* F9-2 plasmid p1-F9 DNA (AP022661.1). Interestingly, an additional blastn search reported a 97% query cover and 98.2% percentage identify match to *B. xylanisolvens* strain H207 plasmid unnamed1. No virulence or antibiotic resistance genes were identified on either plasmid.

The remaining five contigs were identified as belonging to the GB124 genome of 5,093,249 bp with a GC content of 43.87%. A total of 4266 ORFs were predicted of which 72 were tRNA, 1 tmRNA and 2 CRISPR repeat regions (Supplementary Table 6). Two incomplete prophage regions were identified using PHASTER (Arndt et al., 2016). They were 42.5 and 13.2 kbp in size, all features are depicted in Figure 5A. A total of 5 IS elements were identified. However, investigation of the GenBank file and protein sequences revealed only 1 IS element, IS1182 family ISBf3. Genes flanking the IS element had no known function. No *B. fragilis* virulence factors were identified. Resfinder, NCBI and AMRFinderPlus databases revealed two antimicrobial resistance genes (*cepA* and *tetQ*). The *cepA* gene, observed in >90% of *B. fragilis* isolates, encodes the β-lactamase protein and confers resistance to cephalosporins (except cefoxitin) and penicillin (Rogers et al., 1993; Mastrantonio et al., 1996). *tetQ* gene-related resistance is common among *B. fragilis* isolates and encodes a protein that protects the bacterial ribosomes from tetracycline (Rasmussen et al., 1993; Roberts, 1996). Bacterial contigs >200 bp were submitted to GenBank thus omitting 162bp contig from the assembly.

## Discussion

The escape of *B. fragilis* from the gut environment into other parts of the body can result in major pathology, including bacteremia and abscess formation in various tissues. Although phages are a therapeutic option to treat and kill pathogenic *B. fragilis* strains, only three to date had been described and their genomes sequenced. Here, we identify a new highly specific virulent phage that is only able to infect a single host among a panel of *B. fragilis* strains tested. This supports similar finding of ϕ B124-14 which infected 5 out of 15 *B. fragilis* spp. tested (Ogilvie et al., 2012). This narrow host range may reflect extreme niche specialization exhibited by close phylogenetic and evolutionary relationships to gut bacteria (Zaneveld et al., 2010; Ogilvie et al., 2012). The morphological features of the phage identify it as *Siphoviridae.* The absence of any identifiable genes essential for the lysogenic life cycle is consistent with vB_BfrS_23 being a virulent phage. Despite identifying recombinase (CD7) and anti-repressor genes (CD70) which are associated with temperate life cycles, no integrase or excision genes that are essential for lysogenic life cycle were identified. Recombinase and anti-repressor genes have also been identified in ϕB124-14, B40-8 and Barc2635 (Figure 3). The investigators that initially described ϕB124-14 concluded that it was a virulent phage based upon a deviation in GC content between the phage and host, as we have seen between vB_BfrS_23 (38.6% GC content) and *B. fragilis* GB124 (43.87% GC content) (Deschavanne et al., 2010; Ogilvie et al., 2012). Thus, we assume that vB_BfrS23 is a virulent phage that may be a model candidate for human-specific microbial source tracking in contaminated surface and groundwater.

The phage vB_BfrS_23 contains a putative peptidase protein, but lacks any homology to known holin proteins (small membrane proteins) which is not unusual for phages belonging to the *Siphoviridae* family (Hawkins et al., 2008; Duhaime et al., 2011). Double-stranded DNA phages typically lyse host cells using a holin-endolysin system. Active degradation of bacterial peptidoglycan is achieved with a muralytic enzyme or endolysin (Young, 1992; Young and Blasi, 1995). Endolysins accumulate in an active state in the cytosol, the holin proteins bind to the membrane, and the membrane is permeabilized to the endolysin. This leads to the breakdown of murein’s resulting in the cell bursting. All this is time dependent and is programmed into the holin gene (Wang et al., 2000). It appears that the putative peptidase protein resides within an undefined lytic life cycle module in which there may be a holin-endolysin system. Interestingly, a putative thymidylate synthase was identified (CDS11) within the replication and regulation gene module. It is highly conserved across bacterial and mammalian species and shares remarkable structural and functional similarities (Carreras and Santi, 1995; Escartin et al., 2008). The exact function of ThyA within the phage genome is unknown but its additional copies may be of importance for survival of its host by enhancing growth (Stern et al., 2010). No tRNA genes were identified. The genome map highlights only 13 of the 73 predicted coding regions with a putative function, emphasizing the fact that phages are under-characterized.

In comparing the genome of vB_BfrS_23 with that of ϕB124-14, the former is 852 bp larger. Both genomes have genes unique to them that are primarily located around the same gene module and near the cos site, possibly due to recombination events. The Barc2635 genome is 2,021 bp smaller than vB_BfrS_23. There are distinct putative genes present in vB_BfrS_23 that are missing in Barc2635 including CDS 6, 31-34 and 71 hypothetical proteins. The tail fiber protein (CDS 21) is also smaller in Barc2635, B40-8 and ϕB124-14 compared to vB_BfrS_23 by 40 – 483bp (Figure 3). The relatively large tail fiber is consistent with that is seen for *Bacteroides fragilis* phages.

The large terminase subunit and tail fiber phylogenetic comparison shows these genes share homology with other known *B. fragilis* phages. Furthermore, the large terminase subunit is smaller in vB_BfrS_23 (CDS 51) and Barc2635 compared to ϕB124-14. Interestingly the lytic tail fiber genes share higher nucleotide identity with other virulent *B.fragilis* phages compared to the prophage tail fibers, as they appear on another clade. This may be due to the life cycle of the phages and may be an indicator of differences in host range of virulent versus temperate phages. The large terminase subunit shares more identity to ϕB124-14, Barc2635 and B40-8 *B. fragilis* phages (Figure 4A).

The thermal assays provided useful information about vB_BfrS_23. The phage was resilient to the lower temperatures tested (4, 24, 30, 37°C) and at 40 and 45°C an increase in the number of plaques was observed. A key observation is that the phage forms aggregates (Figure 1B) and at higher temperatures (≤ 40 and 45°C) the plaque size was consistently 0.5 mm (Supplementary Figure 7). This characteristic of *B. fragilis* phages showing different plaques sizes and the phage agglomeration or clumping has been reported previously (Keller and Traub, 1974) for *B. fragilis* phages isolated from animal sera (Keller and Traub, 1974). It is possible therefore that the temperature kinetics impacts phage clustering by influencing the binding between phages, potentially mediated by Ig-like or carbohydrate adherence domains (CAD; Sathaliyawala et al., 2010; Barr et al., 2013; Tariq et al., 2015) enabling a more accurate determination of phage numbers in the sample. Alternatively, this could be an artifact of the experimental procedure used to generate samples for EM imaging including the duration of vortexing, the media and need for high phage titers. The vB_BfrS_23 thermal stability characteristics supports work done previously with naturally occurring GB124 phages (Bertrand et al., 2012; McMinn et al., 2014). The first of these studies showed the phages persisted longer at 5°C compared to higher temperatures (20 and 35°C), where few plaques were observed after 7 days (McMinn et al., 2014). Similarly, a meta-analysis of virus inactivation found inactivation occurred faster at temperatures ≥50°C than at <50°C. Adaptation to temperature is important in viral ecology as it influences phage infection, propagation and importantly, viability (Meschke and Sobsey, 2003; Jacquet et al., 2005; Ebdon et al., 2007; Jonczyk et al., 2011).

The exact origin of *B. fragilis* strain GB124 is unknown. It was isolated from untreated wastewater from a treatment plant in South East England and wastewater and impacted surface waters in the United Kingdom, United States, Brazil, and India (Payan et al., 2005; Prado et al., 2018; Wadhwa et al., 2018). Coupled with previously reported existence of a clear human gut-associated eco-genomic signature within the *Bacteroides* phage genomes (Ogilvie et al., 2012, 2013, 2018), it is assumed to be a human gut commensal.

*Bacteroides fragilis* phages when compared with other *Bacteroides* spp. phages have been shown to share little homology (Gilbert et al., 2017). A study looking at ϕBrb01 and ϕBrb02 phages, that are capable of infecting a *Bacteroides* isolate, has shown them to be phylogenetically distant to both ϕB124-14 and ϕB40-8 based on the comparative analysis of the large terminase subunit gene (Gilbert et al., 2017). Similarly, vB_BfrS_23 shares little or no sequence identity to 27 recently published *B. thetaiotaomicron* phages (Hryckowian et al., 2020). Although some CrAssphages such as ϕCrAss001 infect *Bacteroides intestinalis* (Shkoporov et al., 2018), they share little sequence identity to ϕB124-14 and ϕB40-8 (Garcia-Aljaro et al., 2017). Considering that the *B. fragilis* phage presented in this study shares a high sequence homology to other *B. fragilis* phages including ϕB124-14 and ϕB40-8 and thus we can infer that vB_BfrS_23 is also unrelated to other *Bacteroides* spp phages and CrAssphages.

## Conclusion

The isolation and characterization of phage vB_BfrS_23 not only adds to and builds on fledgling phage databases, but it should also facilitate the detection other *Bacteroides* phages in human fecal metagenomes. As both the bacterial host and the new phage reported here are sourced from municipal wastewater they have considerable potential as, (1) highly specific novel therapeutic agents, (2) as tools for testing the efficacy of water and wastewater reuse technologies (spiking studies), and (3) as molecular or metagenome-based Microbial Source Tracking genetic marker for identifying human fecal transmission pathways in contaminated water and food (McMinn et al., 2014; Dias et al., 2018).

## Data Availability Statement

The datasets presented in this study can be found in online repositories. The names of the repository/repositories and accession number(s) can be found below: https://www.ncbi.nlm.nih.gov/genbank/, SAMN14 843706 SRA; https://www.ncbi.nlm.nih.gov/genbank/, SRX8283257; https://www.ncbi.nlm.nih.gov/genbank/, SRX SRX828326; https://www.ncbi.nlm.nih.gov/genbank/, SRX8275163; https://www.ncbi.nlm.nih.gov/genbank/, SRX8275162.

## Author Contributions

SC and MT conceived and designed the experiments. MT, FN, JE, LH, and SC wrote the manuscript. SC supervised the research. MT, FN, RH, CB, and LH executed the experimental work. MT, FN, LH, MC, TW, JE, and SC carried out the data interpretation. All authors revised, read, and approved the final manuscript.

## Conflict of Interest

The authors declare that the research was conducted in the absence of any commercial or financial relationships that could be construed as a potential conflict of interest.

## Footnotes

1 https://nanoporetech.com

2 https://github.com/rrwick/Porechop

3 https://github.com/tseemann/abricate

4 http://www-is.biotoul.fr

5 https://ccb-microbe.cs.uni-saarland.de/plsdb/

6 https://blast.ncbi.nlm.nih.gov/Blast.cgi

## Abbreviations

BPRM: *Bacteroides* phage recovery medium
CDS: coding sequence
ORF: open reading frame.
