# Supplementary material for: Genome Characterization of a Novel Wastewater Bacteroides fragilis Bacteriophage (vB_BfrS_23) and its Host GB124
Source: Front Microbiol. 2020 Oct 23;11:583378. doi: 10.3389/fmicb.2020.583378 (PMC7644841; doi:10.3389/fmicb.2020.583378)
Supplement: Supplementary file 1 [file Data_Sheet_1.PDF]

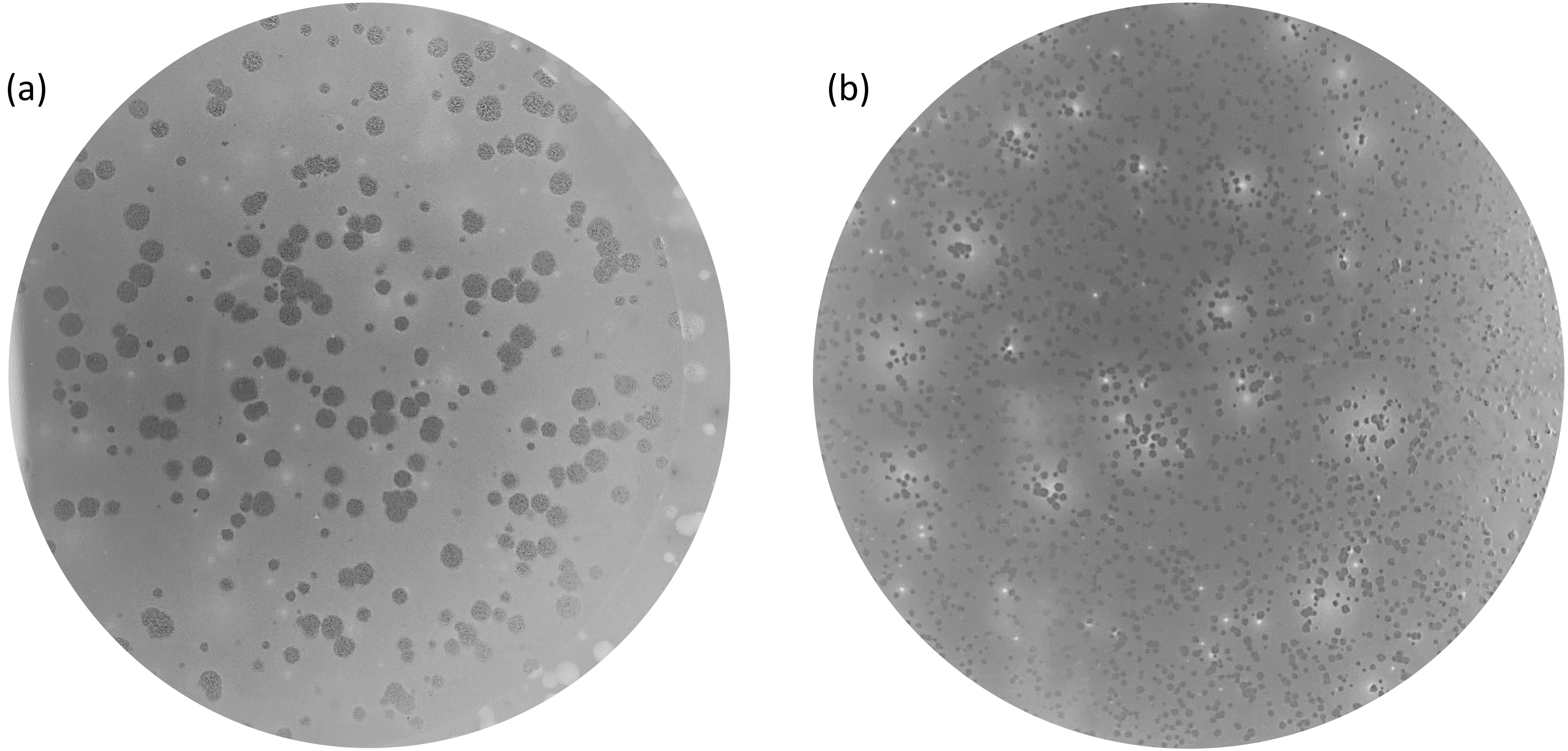

Figure S7: vB_BfrS_23 plaque morphologies. The bacteriophage vB_BfrS_23 was incubated with its host GB124 at different temperatures using a double agar method (BPRM, 0.35% w/v). (a) morphology of plaques obtained after incubating with phage dilution of 10^{-4} at 37 °C for 15 min showing uniform plaque morphologies of up to 2mm diameter. (b) morphology of plaques obtained after incubating with phage at 10^{-3} dilution at 45 °C for 30 min showing smaller uniform plaques of approximately 0.5mm diameter.
